# Supplementary material for: Strong hydroclimatic controls on vulnerability to subsurface nitrate contamination across Europe
Source: Nat Commun. 2020 Dec 9;11:6302. doi: 10.1038/s41467-020-19955-8 (PMC7725821; doi:10.1038/s41467-020-19955-8)
Supplement: Supplementary file 1 — Supplementary Information [file 41467_2020_19955_MOESM1_ESM.pdf]

Supplementary Information for “Strong hydroclimatic controls on vulnerability to subsurface nitrate contamination across Europe” by Kumar et al

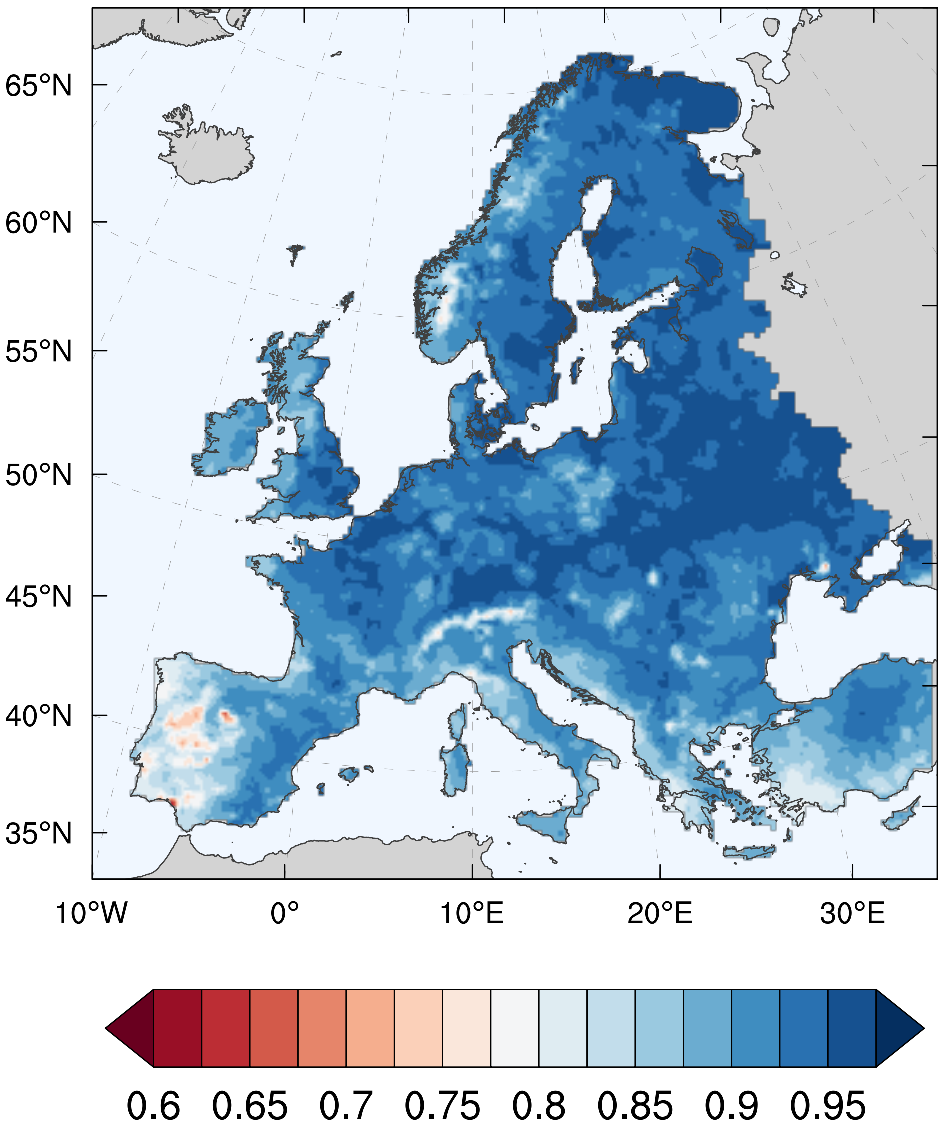

Supplementary Figure 1: Spatial map depicting the correlation between the daily time-series of the median and interquartile ranges of the travel time distributions (TTDs) for the simulation period.

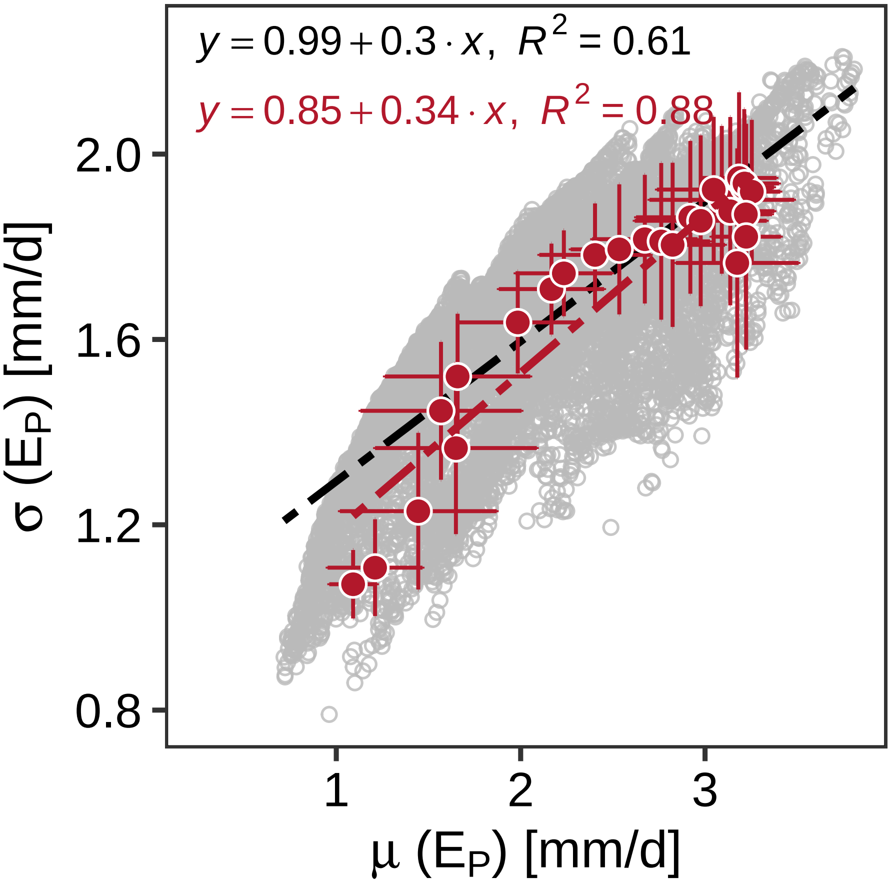

Supplementary Figure 2: Scatter plots depicting the correspondence between the mean and standard deviation of the daily potential evapotranspiration, i.e., $\mu(E_P)$ vs. $\sigma(E_P)$ – based on data of all grid cells located within the study area. The daily E_P is derived based on the Hargreaves and Samani method[1] using the site-specific daily averaged, maximum and minimum air temperature values for the period 1950–2015. Overlaid upon the raw data points (in gray) are the scatter points corresponding to bin estimates (in red) as mean and one standard deviation values of grouped data for every ϕ interval of 0.15, similar to Figure 2 in the main text. Also shown are the best fitted regression lines (equations and R^2) for both raw and binned (average) values.

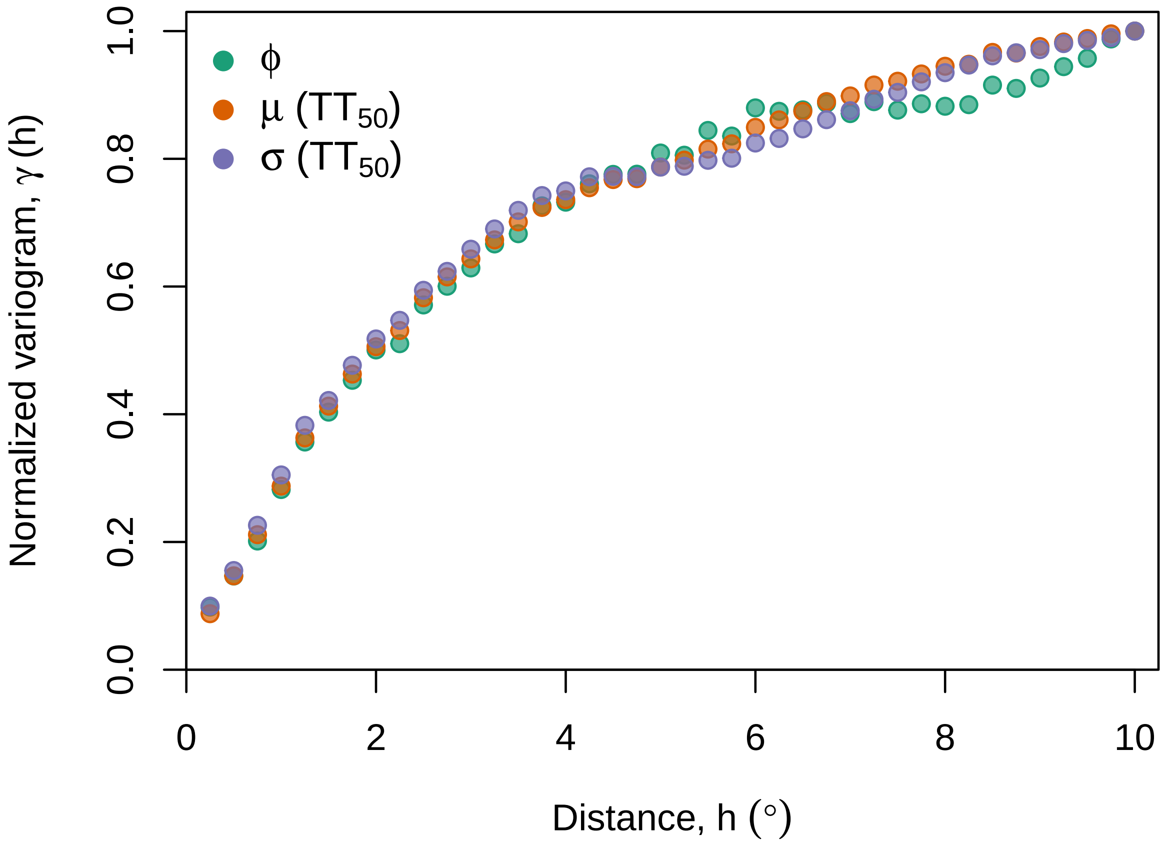

Supplementary Figure 3: Semivariogram summarizing the spatial structure of the aridity index (ϕ) and the temporal mean and standard deviation of the daily median travel times – $\mu(\mathrm{TT}_{50})$ and $\sigma(\mathrm{TT}_{50})$ – observed across the study domain based on the underlying data of Figures 2a,b,c in the main text. Semivariogram estimates of each variable are normalized to ensure comparability among different variables.

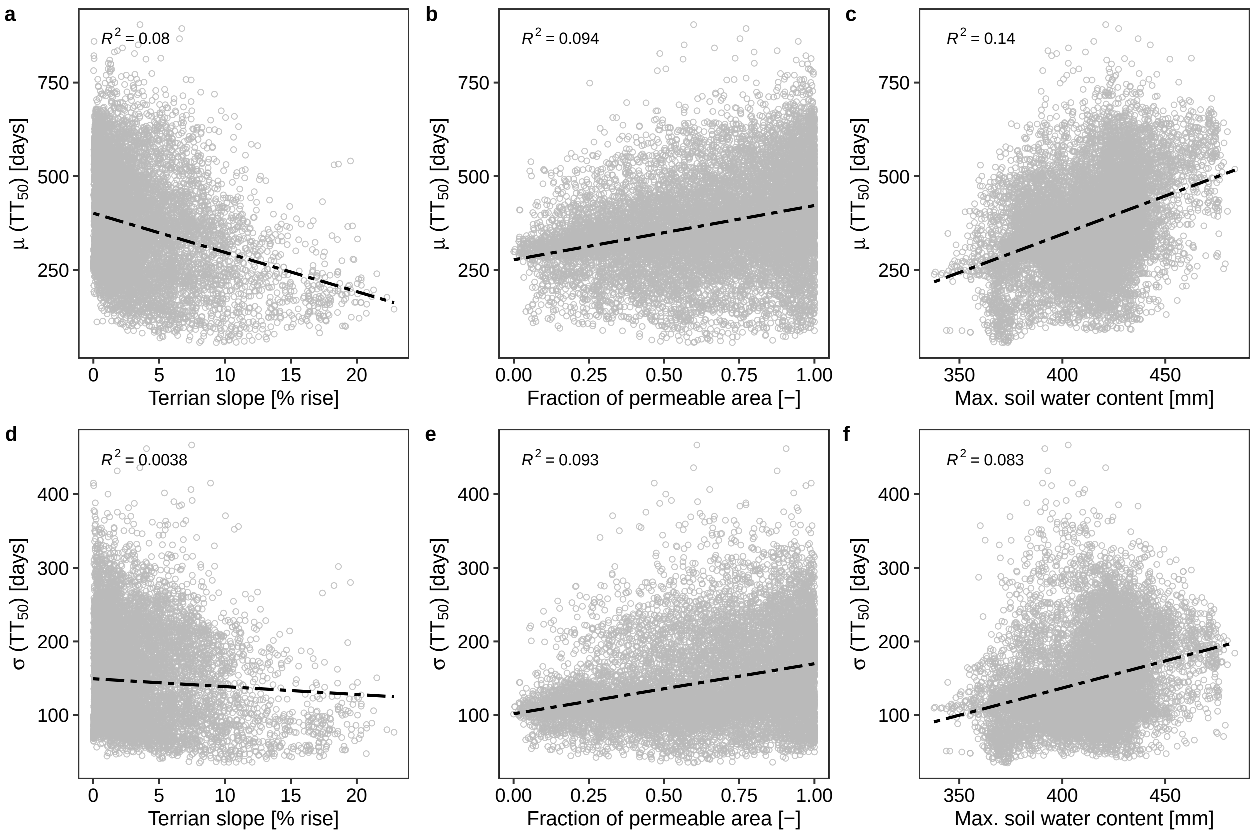

Supplementary Figure 4: Scatter plots depicting the correspondence between grid-specific landscape attributes (i.e., average terrain slope, fraction of permeable areas and maximum root-zone soil water content) and travel times statistics (i.e., temporal mean $\mu(\mathrm{TT}_{50})$ and standard deviation $\sigma(\mathrm{TT}_{50})$ of the daily median travel times (TT_{50}) whose spatial patterns are shown in Figure 2a,b in the main text). The respective best fit regression line (in dashed black line) and correlation coefficient estimate (R^2) are also shown in each plot. Permeable areas refer to all types of landcover except the urban and forested areas. Maximum soil water content is the product of porosity and root-zone depth, where the former is estimated via a set of pedo-transfer functions in a multiscale parameter regionalization framework[2–4].

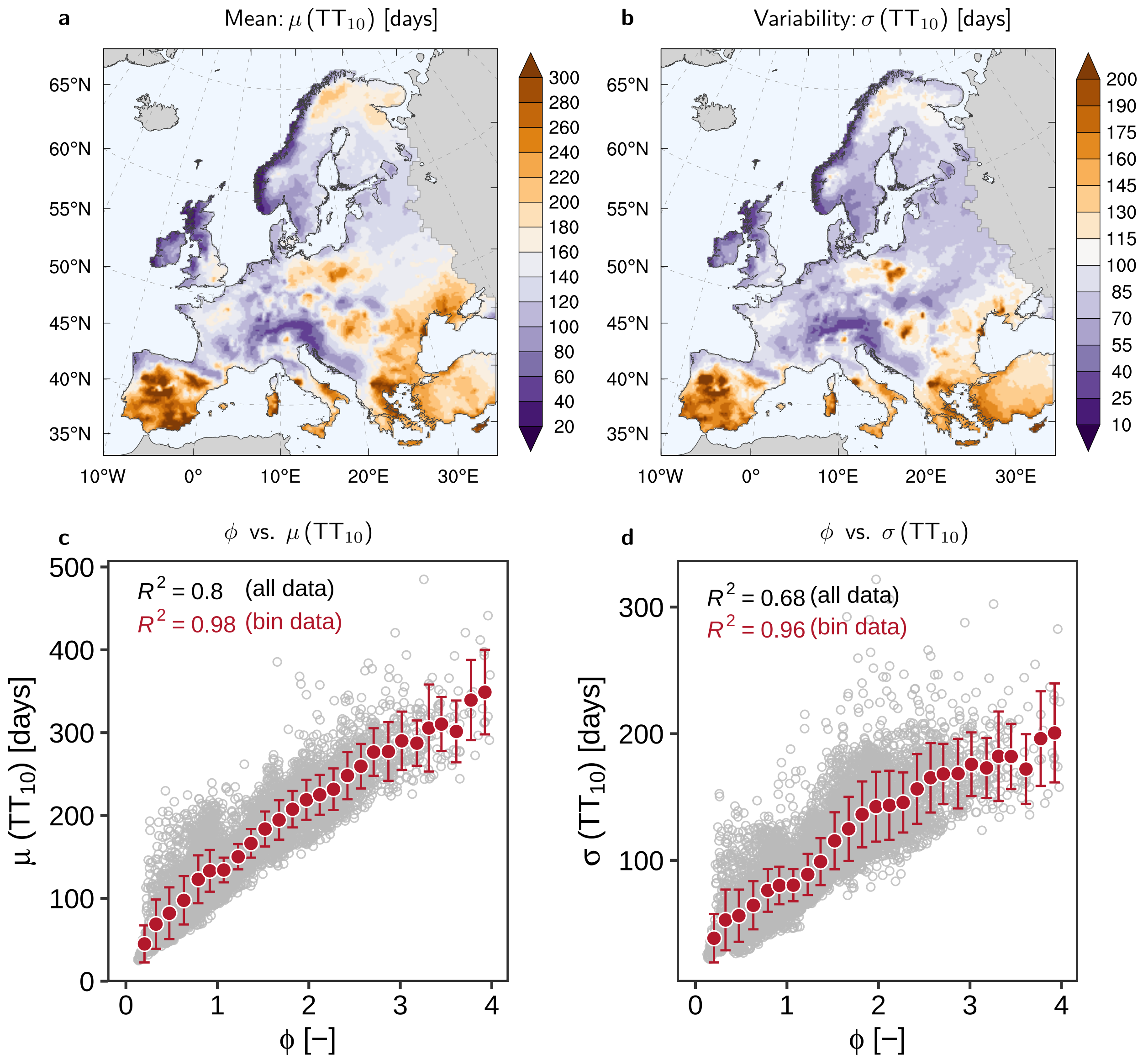

Supplementary Figure 5: Similar to Figure 2 of the main text, spatial maps (a,b) show the temporal mean $\mu(\mathrm{TT}_{10})$ and standard deviation $\sigma(\mathrm{TT}_{10})$ of the daily 10th percentile estimates of travel time distributions (TTDs). The bottom scatter plots (c,d) show the correspondence of the grid-specific $\mu(\mathrm{TT}_{10})$ and $\sigma(\mathrm{TT}_{10})$ with the respective aridity index (ϕ) along with the correlation coefficient estimate (R^2). On each of the scatter plot, along with point-wise cloud data there are also the corresponding bin estimates given as mean and one standard deviation values of grouped data for every ϕ interval of 0.15.

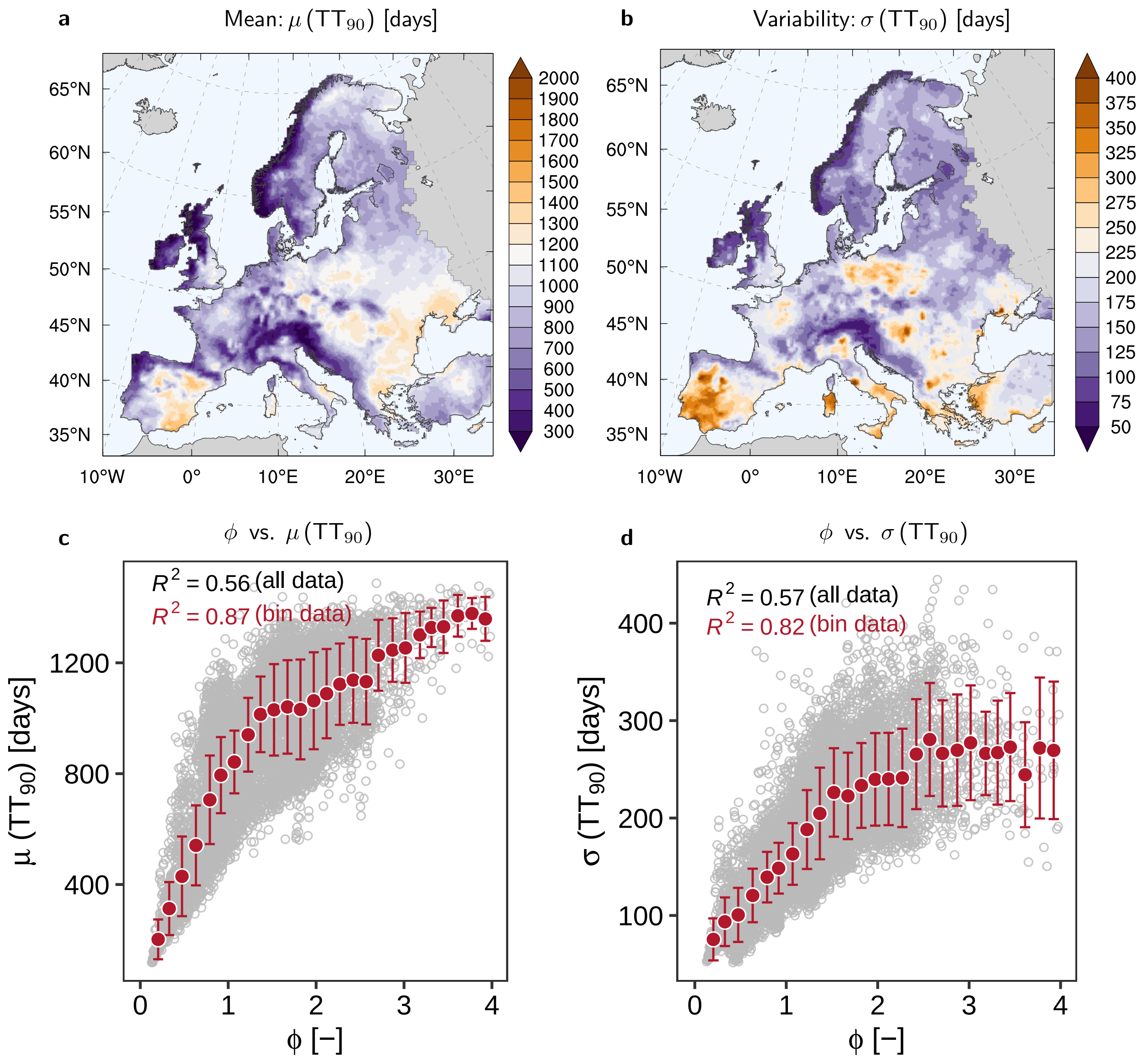

Supplementary Figure 6: Same as Figure 5, but for the daily 90th percentile estimates of travel time distributions (TTDs).

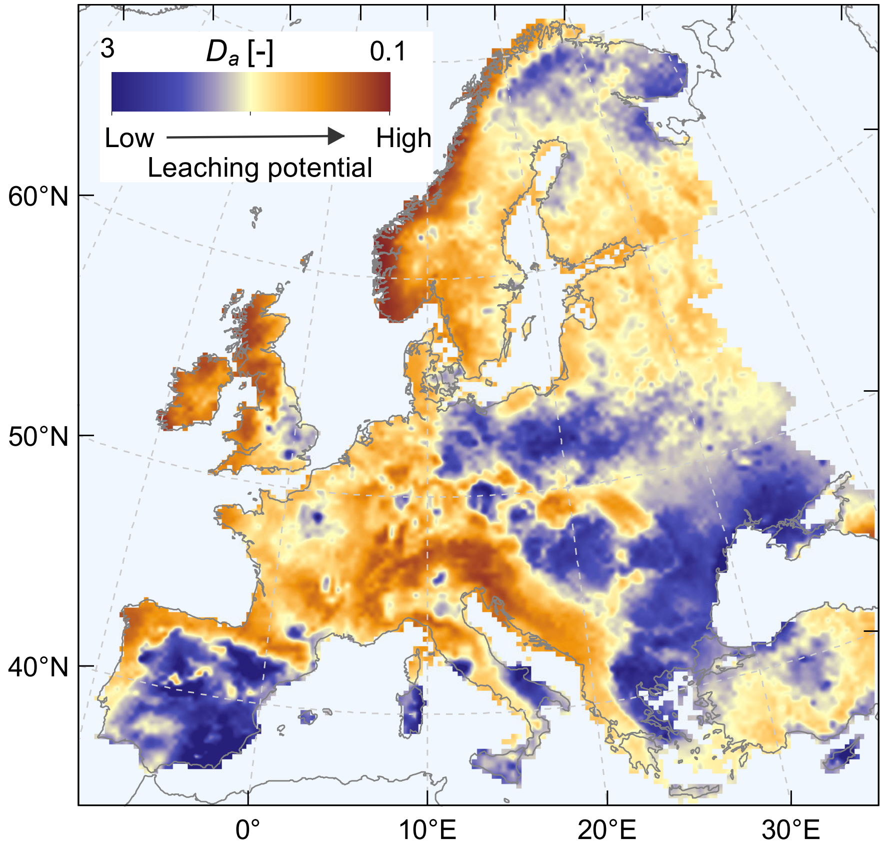

Supplementary Figure 7: Spatial variability of the Damköhler number (D_a) depicting the soil nitrate leaching potential map across Europe, computed in our study. The underlying information on D_a is same as Figure 3b of the main text, but plotted here on a continuous scale with the color coding being adapted to a leaching risk potential map published by the European Commission; EC[5] (taking into account for a colorblind-friendly palette). The lower D_a values represent the areas vulnerable to subsurface nitrate leaching and vice-versa (see the main manuscript for more details). One may refer to the cited report for the EC published leaching map. A high degree of spatial similarity between both maps can be observed in terms of low and high leaching areas especially in the Central and Western European regions, as well as in Mediterranean ones. There are also differences between both maps mostly in east European regions. However reconciling these differences are not possible because the underlying data-sets and methodology used in creating the EC map are not provided. Nevertheless there is a large scale consistency in vulnerable/non-vulnerable areas depicted in both maps and the regions with differences could be focus of targeted future studies employing local information/datasets.

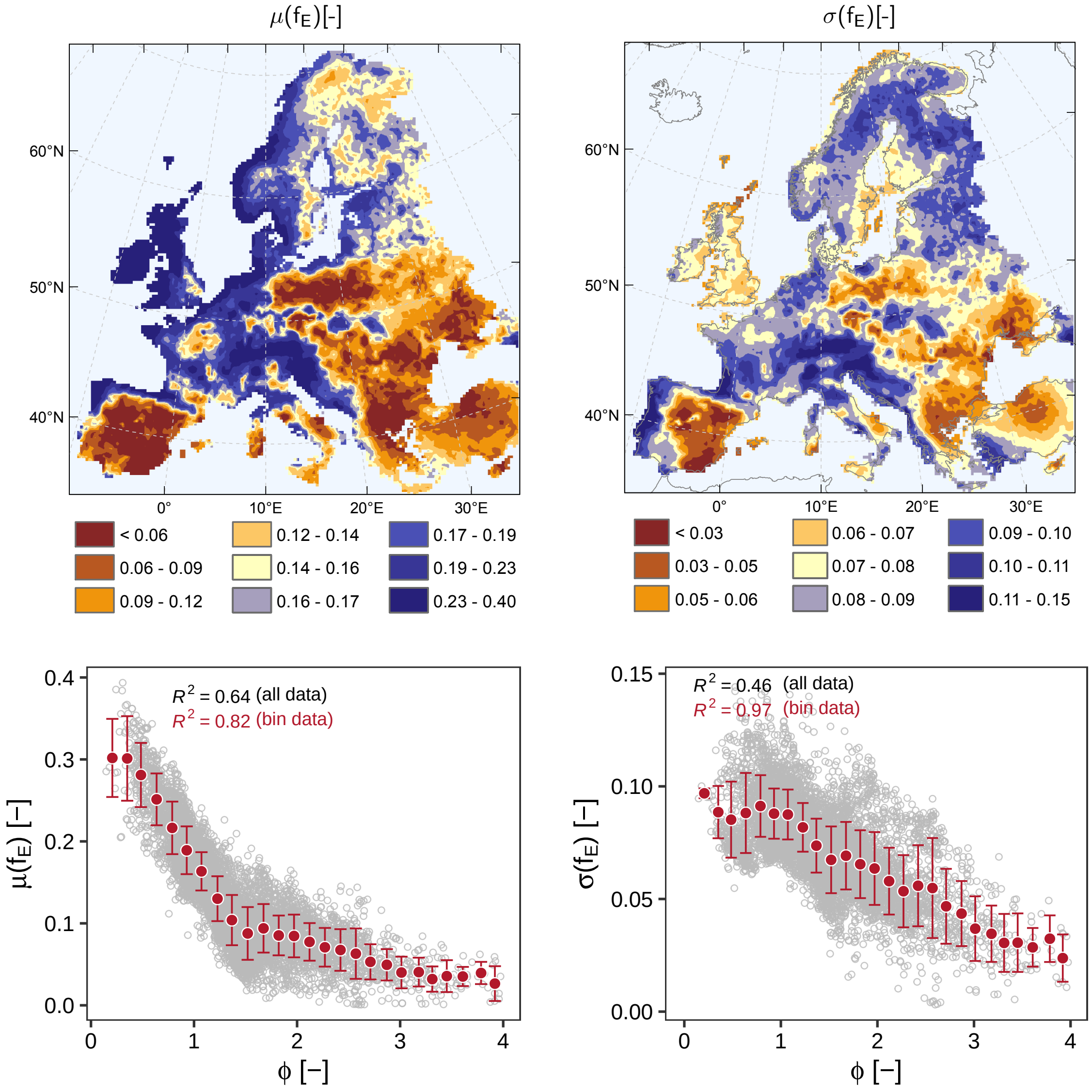

Supplementary Figure 8: Europe-wide variability of the daily environmental factors (f_E), affecting the root-zone denitrification timescale across the study domain. Shown are the mean (μ) and the standard deviation (σ) of the daily f_E values. Below scatter plots show the correspondence of the grid-specific f_E estimates with the corresponding aridity index (ϕ) values along with the correlation coefficient estimate (R^2). In the scatter plot, along with point-wise cloud data there are also the corresponding bin estimates given as mean and one standard deviation values of grouped data for every ϕ interval of 0.15 (similar to Figure 2 in the main text).

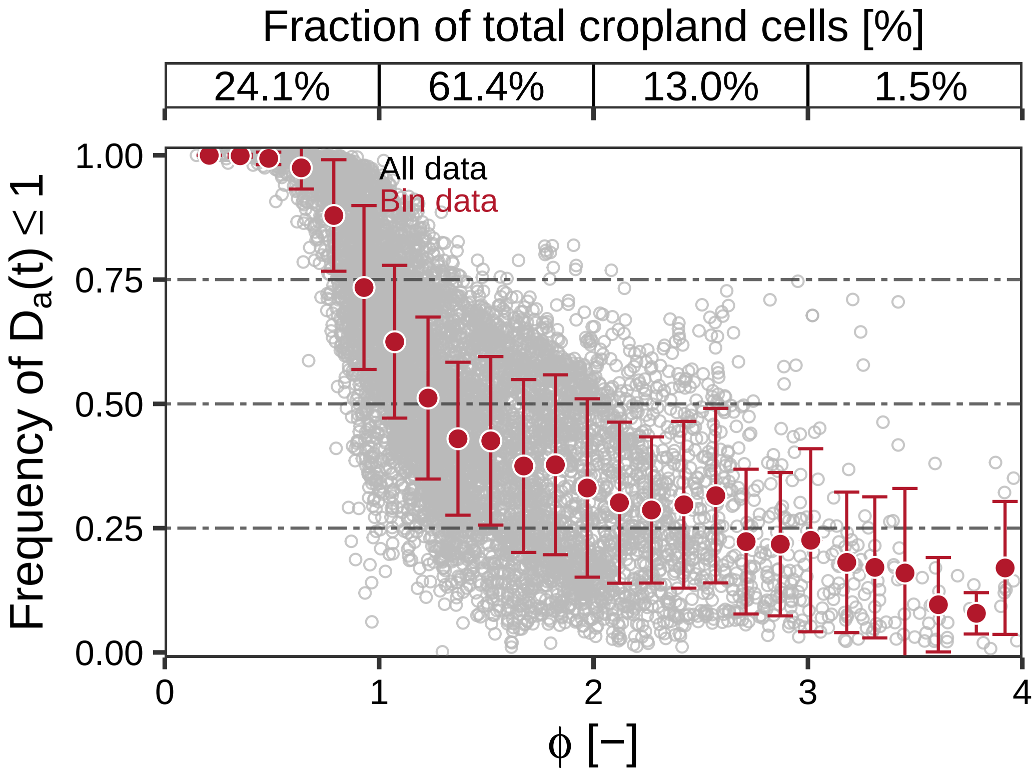

Supplementary Figure 9: Scatter plot depicting the correspondence of the grid-specific frequency values of the daily $\mathrm{D_a}(t) \leq 1$ and the respective aridity index (ϕ) for the cultivated areas across Europe. On the scatter plot, along with point-wise cloud data there are also the corresponding bin estimates given as mean and one standard deviation values of grouped data for every ϕ interval of 0.15. The underlying values of the frequency estimates correspond to Figure 3d of the main text. Also shown in the top are the fractions of cropland cells falling within different aridity (ϕ) classes.

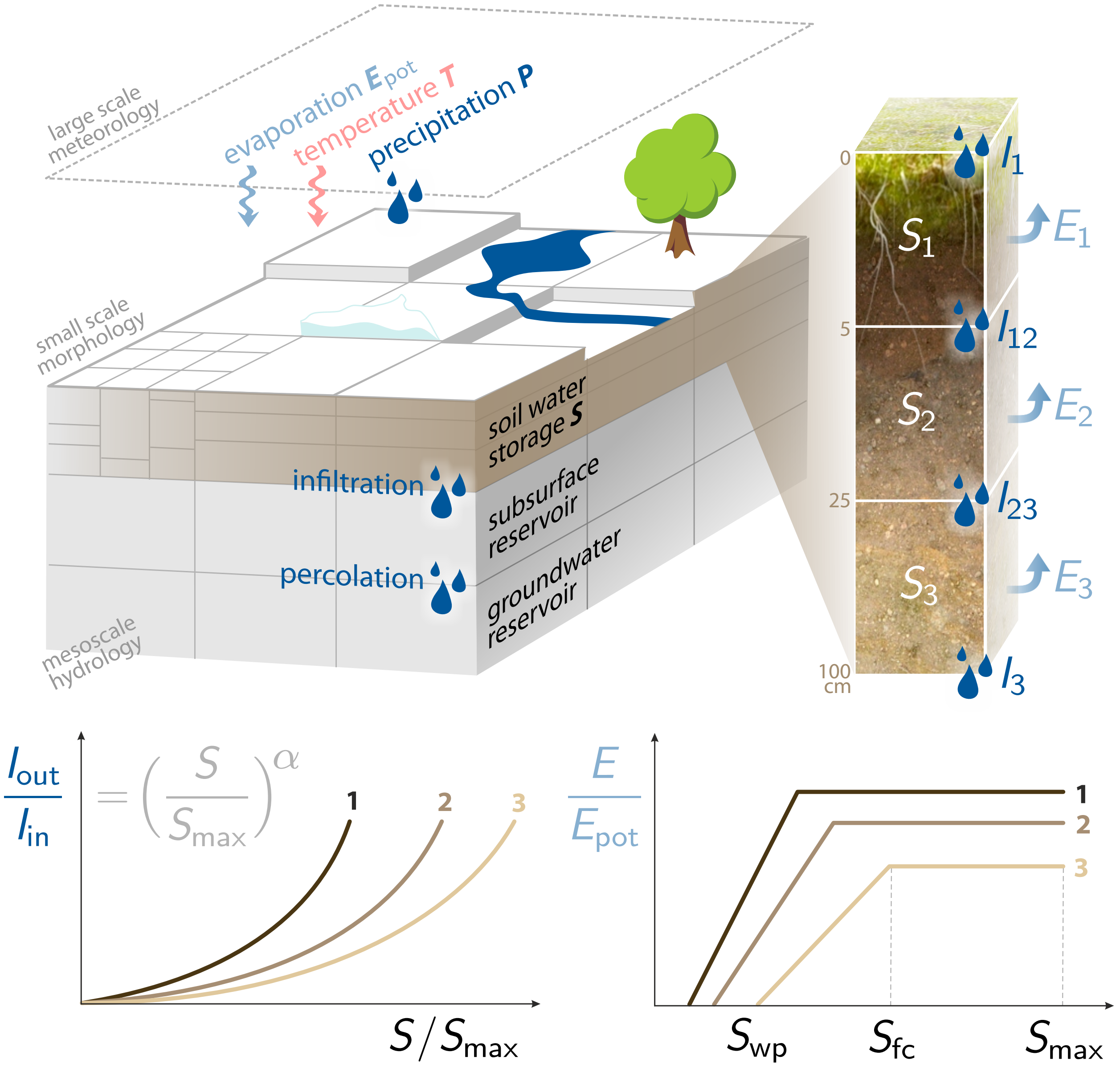

Supplementary Figure 10: Schematic of the key components of the mesoscale Hydrologic Model (mHM) with parts highlighting the root-zone soil moisture processes in three layers. S, I, and E denote soil-water content, infiltration, and evapotranspiration, respectively. S_{wp}, S_{fc}, and S_{max} denote the soil water content at wilting point, field capacity and saturation, respectively – determined through the multiscale parameter regionalization scheme[2–4]. The below panels show the conceptualization of the processes determining the water fluxes leaving the given soil layer (I_{out} and E) as a function of the respective layer's soil-water content, the degree of non-linearity (α), and the potential evapotranspiration (E_{pot}).

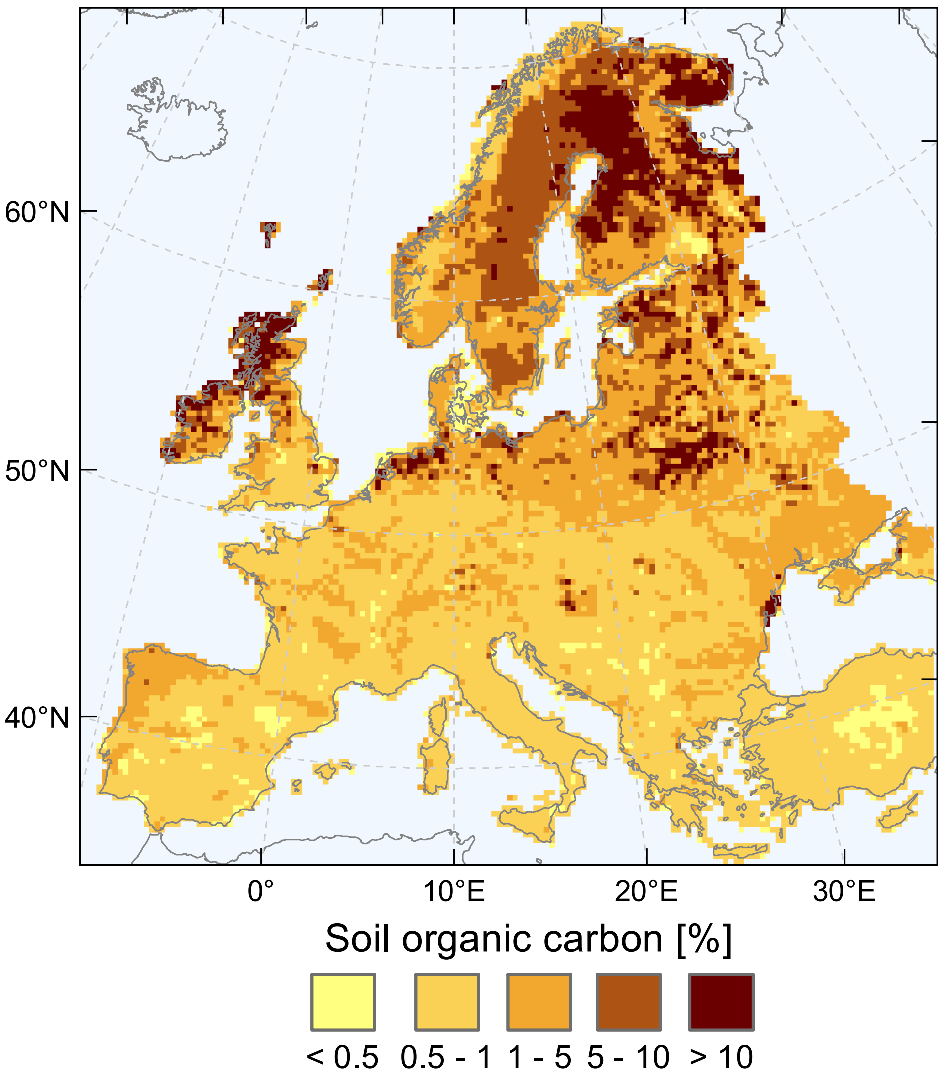

Supplementary Figure 11: Spatial distribution of the soil organic carbon content in the root-zone soil layer estimated based on the Harmonized World Soil Database[6].

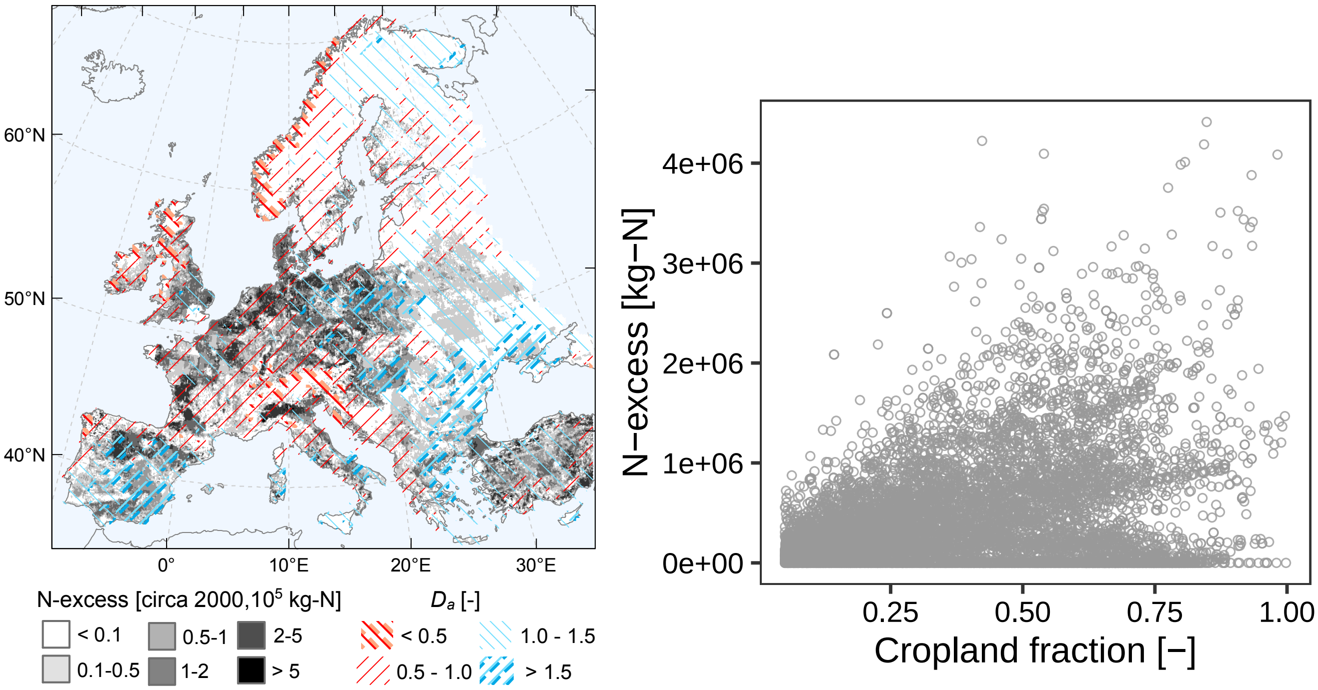

Supplementary Figure 12: Spatial distribution of the N-surplus (or N-excess) estimates for major crops around the year 2000 based on a prior study[7]. Similar to Figure 3b of the main text, the overlain hatches are the varying Damköhler $(\mathrm{D_a})$ numbers corresponding to the averaged $\mathrm{TT_{50}}$ and effective $\langle RT_{50} \rangle$ value of 1 year. Right panel shows the scatter plot between grid-specific cropland fractions and the N-surplus estimates.

Supplementary Note 1

The study is conducted across Europe which is characterised by a diverse range of climatic and landscape conditions. A range of publicly free datasets are assembled to establish the hydrologic model mHM. This consists of the physiographical datasets, which mainly include Digital Elevation Model (DEM), river network, soil textural properties, and landcover and geological characteristics. The data-processing and the establishment of the mHM-MPR framework adopted here follow a framework of the recently concluded project "End-to-end Demonstrator for improved decision-making in the water sector in Europe"(EDgE[8]). A brief overview of underlying datasets used in our study is provided in Supplementary Table 1 and the processing steps are detailed below.

The DEM data is processed to extract information on flow accumulation, flow direction, terrain slope, and aspect. Soil textural properties include the sand, clay and bulk density information for different soil horizons. The land cover characteristics include distinction between different land cover classes (e.g., forest, cropland, urban areas) and the long-term monthly mean leaf area index (LAI). Four scenes of landcover map are available from the CORINE dataset corresponding to the year 1990, 2000, 2006, and 2012. Landcover information outside of the CORINE domain is augmented with the Global land cover data (ESA) for the year 2005. Model simulations prior to 1990 use the landcover information of the year 1990. Soil textural information (e.g., horizon specific sand and clay contents, bulk density, etc.) are derived from the globally available Harmonized World Soil

Database (HWSD). Finally the geological characteristics show the distinction between different geological classes, with different aquifer properties. Notably, these datasets are available at different spatial resolutions (e.g., DEM at 25 m, soil map at 1 km, landcover at 250 m), they are mapped on a common spatial resolution of 500 m $\times$ 500 m. These fine scale datasets allow to account for the sub-grid variability of the landscape characteristics through the multiscale parameter regionalization technique implemented in the mHM modeling framework[2–4,9].

The meteorological forcings for the mHM consist of the daily fields of precipitation, and average, maximum and minimum air temperatures at 0.25° $\times$ 0.25° resolution for the period 1950–2015. These datasets are acquired from the European Climate Assessment and & Dataset project (E-OBS, v13.0)[10]. These fields are created using the external drift Kriging interpolation technique based on ground-based observation networks. The daily fields of potential evapotranspiration are derived based on the Hargreaves and Samani method[1] at the same spatial resolution (0.25° $\times$ 0.25°).

We used the multivariate information on water fluxes and states available at different resolutions to assess the skill of modeled variables. These include the streamflow measurements from more than 300 gauging stations located across Europe, and are available from the Global Runoff Data Centre (GRDC). The catchment size varies approximately between 1000 km^2 and 800,000 km^2, with a median area of approximately, 5000 km^2. The availability of streamflow data varies from station to station with the median record

length of approximately 45 years between the period 1950–2015.

The second set of evaluation dataset consists of the 0.5° gridded ET dataset[11] derived from the FLUXNET observations for the period 1982–2011. This dataset is created using a machine learning approach consisting of a model tree ensembles (MTE) and is based on the upscaling of observations of biosphere-atmosphere fluxes of carbon and energy from eddy covariance flux tower sites[11].

The final set of evaluation consists of a gridded product of the total water storage (TWS) anomaly at 1° spatial resolution, retrieved by the Gravity Recovery and Climate Experiment (GRACE-RL05)[12]. Three different products of the GRACE based TWS anomaly fields are available from three processing centers: GFZ (Geoforschungs Zentrum Potsdam, Germany), CSR (Center for Space Research at University of Texas, USE) and JPL (Jet Propulsion Laboratory, USA). We used the ensemble mean of the three available GRACE-anomaly products for the model evaluation as it is considered as one the most effective way to reduce noise in the gravity field within the available scatter of the three solutions[13].

The mHM-MPR modeling framework is established across the study domain at 0.25° spatial resolution and daily timescale, using the above datasets and the parameterization established in previous studies[2,3,8,14]. The chosen spatial and temporal resolutions are consistent with the availability of meteorological datasets (E-OBS, v13.0)[10]. Following previous studies[14,15], the model warm-up is configured by first running it for the entire

simulation period (1950–2015), and then the modeled states at the end of the simulation period are taken as initial conditions for the subsequent simulations. To further minimize the influence of initial conditions, the first five years of the model simulations are discarded from further analyses. Additionally we run the model at 0.5° and 1° spatial resolutions for the evaluation against evapotranspiration and terrestrial water storage anomaly, respectively. We conduct these runs with a similar setting of the model parameterizations using a multiscale modeling feature of the mHM-MPR framework[8,14].

We conduct the Europe-wide simulations of hydrologic fluxes and storages focusing on synthesising the large-scale transport behavior and providing assessment of subsurface vulnerability to (excess) nitrate leaching through soil. Our model simulations represent hydrologic behaviors occurring within the terrestrial compartment - near and below Earth surface. First order changes in hydrological processes due to human intervention to terrestrial landscapes are implicitly/explicitly represented in the model, as for example by an explicit accounting of the space/time variability of vegetation and landcover dynamics, or the parameterization of interflow components which implicitly accounts for landscape attributes (varying slopes and overlying vegetation activities) that affect the near-surface drainage and flow components. With regard to the latter example, the model does not explicitly account for any physical intervention as for example in case of tile-drainage that promotes the transport activities. We expect that such direct interventions would have minimal effect on the overall modeling results presented in this study given the fact that our assessment is focused on the transport processes within the root-zone soil, and the

tile-drainage infrastructures are generally installed below the rooting depth (as e.g., detailed in the FAO Irrigation and Drainage Paper-62[16]. Another important consideration in the arable areas is the provision of irrigation to supplement the deficit of required crop water demand during dry periods. Across the European arable landscapes, according to the EUROSTAT[17] report in 2005, 10.1% of utilised agricultural area in the EU was irrigable but only 6.8% was actually irrigated; and over the time between 2005 and 2016 these have even decreased by 3.5% for the irrigable areas and irrigated areas by 6.1%. There are however regional variations and Spain (15.7%) and Italy (32.6%) have the largest shares of irrigable areas in the agricultural areas[17]. We perform a controlled simulation to analyze the impact of irrigation on resulting transport behaviors. To this end, we adapt an irrigation water demand scheme of the well-established PCR-GLOBWB model[18,19] and contrast the model simulation results of the irrigation (Irr) versus no-irrigation (No-Irr) scenarios. Our simulation results shown in Supplementary Figure 13 confirm that large part of the cultivated area across Europe show minor differences in simulated hydrological fluxes (evapotranspiration ET or leached water I from the soil profile) due to irrigation activities. For example, the changes in evapotranspiration across 95% of cropland cells is less than 50 mm/y. Only few cropland cells ($\approx$ 1.8%) with heavy deployment of irrigation in regions of Spain and east European countries exhibited changes in ET of more than 100 mm/y. Similarly the majority of cropland cells ($\approx$ 98%) show changes in I of less than 50 mm/y between Irr and No-Irr simulations runs. These simulation results of changes in ET and I between irrigation and no-irrigation runs provide plausible scenarios

considering that farmers will irrigate when atmospheric demand is high and as much such that plant can take up most of the (irrigated) water and relatively smaller portion will loss or drain to deeper subsurface. Next we analyze the effect of irrigation on the resulting transport dynamics and our simulation results indicated that increased water availability due to irrigation resulted in reduced transport times (Supplementary Figure 14). However, the majority of the cropland cells ($\approx$ 96%) exhibited changes in the median travel times (TT_{50}) of less than a month; and only few cropland cells ($<$ 1%) showed changes in TT_{50} of more than 2 months (Supplementary Figure 14). In terms of relative changes in TT_{50} with respect to the No-Irr scenario runs, almost 70% of the cropland cells show no changes in TT_{50} and vast majority ($\approx$ 95%) showed a minimal change ($\leq$ 5%). Notable changes ($>$ 10%) in TT_{50} due to irrigation were seen in only 0.7% of the cropland cells (Supplementary Figure 14). In conclusion, these simulation results demonstrate that irrigation activities have a very limited impact on the inferred large-scale patterns of transport dynamics conducted at the European scale.

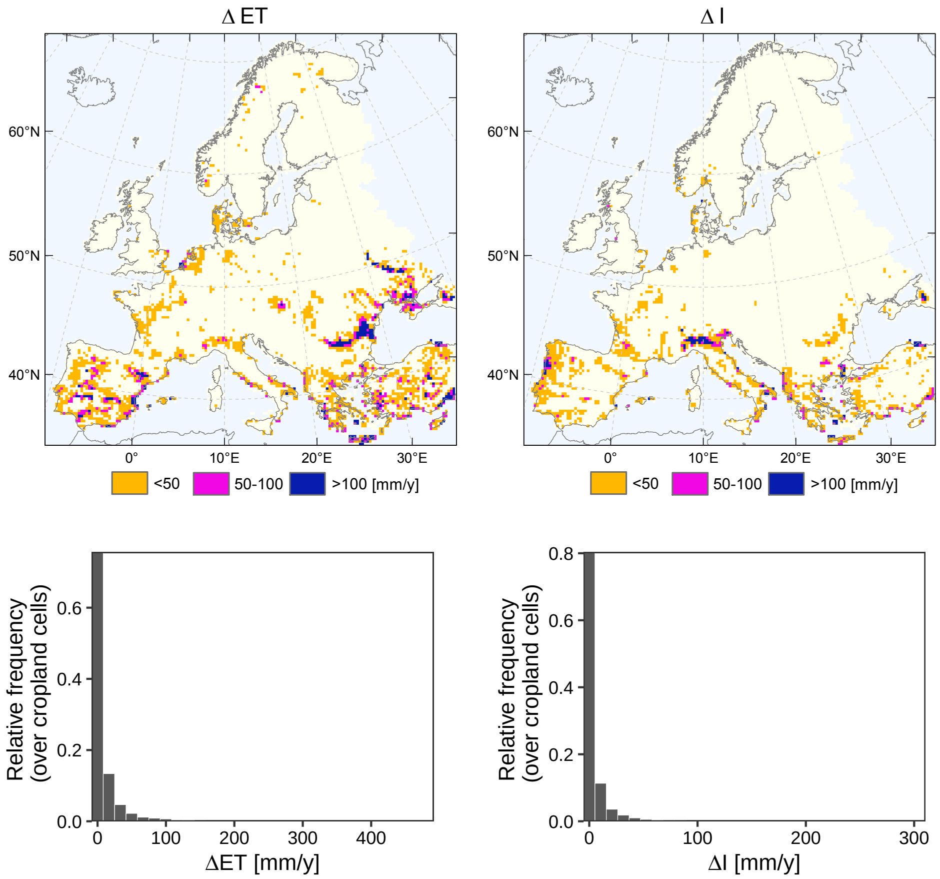

Supplementary Figure 13: Spatial distribution of changes in average evapotranspiration (ΔET) and leached water (ΔI) from the soil profile between the model simulations of irrigation and no-irrigation scenarios. Bottom panels depict the corresponding histogram of changes over the cropland cells. Note that both fluxes show an increase in the irrigation scenario compared to the no-irrigation scenario.

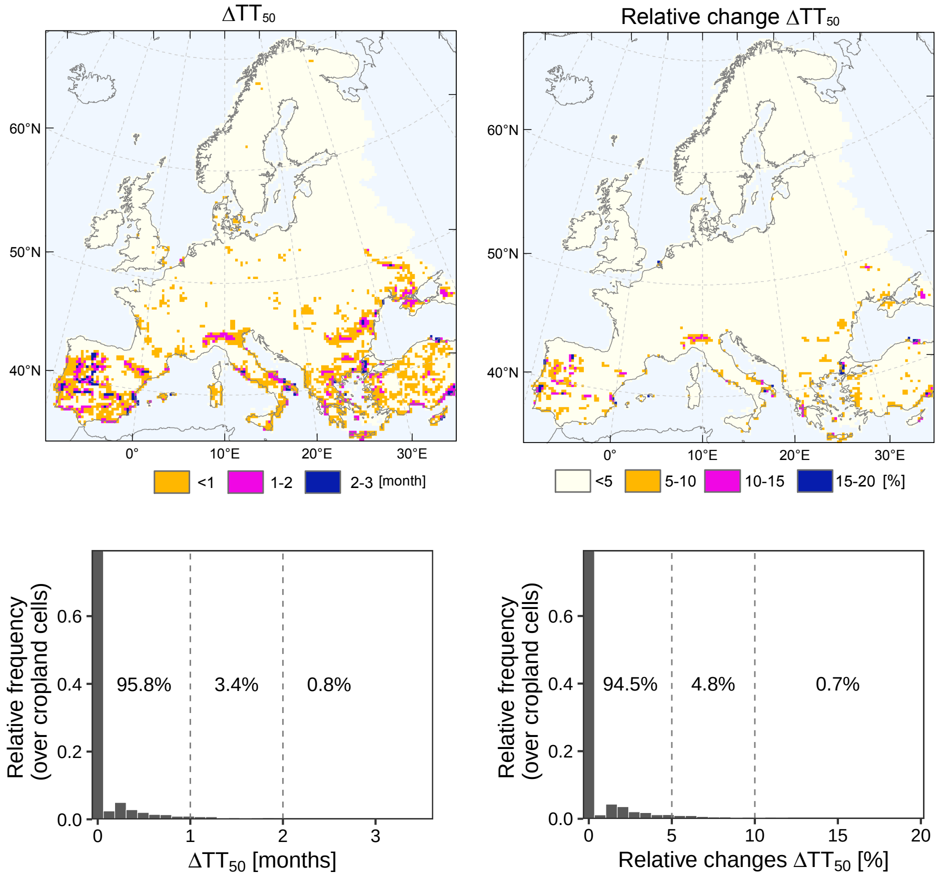

Supplementary Figure 14: Spatial distribution of changes in mean hydrologic transport time scales (median travel time; ΔTT_{50}) between the model simulations of no irrigation and irrigation scenarios. Shown in the right are the relative changes in TT_{50} with respect to base estimates of the no-irrigation scenario. Bottom panels depict the corresponding histograms of changes in ΔTT_{50} over the cropland cells. Note that the median travel times (TT_{50}) are reduced in case of the irrigation scenario (i.e., signs here should be interpreted opposite compared to the changes depicted in Supplementary Figure 13 for the simulated fluxes).

Supplementary Table 1: List of main input and evaluation datasets used for the model set-up over the study domain.

**Variable**	**Description**	**Reference**
Meteorological forcings	Daily E-OBS product at 0.25° resolution	ECA&D[a][10]
Terrain characteristics (e.g., slope, aspect, flow direction and accumulation)	Joined EU-wide and Global (GOTOP30) Digital Elevation Model (DEM)	EEA[b], USGS[c]
River and Catchment Database	River and catchment shapes (CCM-v2.1)	EC-JRC[m]
Land cover classes (e.g., major classes: forest, permeable, impervious cover)	CORINE[d] and GlobCOVER v2 land cover at 250 m resolution	EEA[c], ESA[k]
Leaf Area Index (LAI)	Mean monthly LAI at 5 km	GIMMS[l]
Soil textural properties (e.g., sand and clay)	30 arc-second raster	HWSD[e][6]
Geological classes	IHME[f]; hydrogeological map of Europe	BGR[g]
Streamflow data	Daily observed streamflow	GRDC[h]
Evapotranspiration (ET)	Gridded product of 0.5° resolution	FLUXNET[11]
Terrestrial Water Storage (TWS) anomaly	Gridded product of 1° resolution	GRACE[j][12,20]

[a] ECA&D: European Climate Assessment & Dataset; http://www.ecad.eu

[b] EEA: European Environment Agency; http://www.eea.europa.eu

[c] USGS: U.S. Geological Survey; https://lta.cr.usgs.gov/GTOPO30

[d] CORINE: Coordination of Information on the Environmenthttp://land.copernicus.eu

[e] HWSD: The Harmonized World Soil Database; https://webarchive.iiasa.ac.at

[f] IHME: International Hydrogeological Map of Europe

[g] BGR: Federal Institute for Geosciences and Natural Resources; https://www.bgr.bund.de

[h] GRDC: Global Runoff Data Centre; http://www.bafg.de/GRDC

[j] GRACE: Gravity Recovery and Climate Experiment

[k] ESA: European Space Agency; http://due.esrin.esa.int/page_globcover.php

[l] GIMMS: Global Inventory Modeling and Mapping Studies; http://staff.glcf.umd.edu

[m] Joint Research Centre; http://ccm.jrc.ec.europa.eu

Supplementary Note 2

The mHM modeling framework used here has been extensively evaluated in several recent studies for its skill in reproducing the observed spatio-temporal dynamics of water fluxes and storage components[3,8,14,15,21–25] . Here we provide a brief overview on the model skill for capturing the observed dynamics of streamflow, evapotranspiration and terrestrial water storage anomaly (TWS), based on the datasets described in the Supplementary Note 1.

Overall the model is able to capture the observed dynamics of streamflow reasonably well, as demonstrated over a range of 325 river basins located across Europe and having different characteristics (Supplementary Figure 15). The correlation between observed and simulated streamflows is more than 0.90 (0.82) for 50 % (75 %) of the basins (Supplementary Figure 15a). Relative bias estimated between observed and simulated mean streamflows is also within acceptable limits, as the median relative bias is 2 %, and 50 % of the basins exhibit a relative bias value between $\pm$ 10 % (Supplementary Figure 15b). A relatively poorer model skill is observed in basins which are influenced by a comparatively larger degree of human activities (e.g., dams and reservoirs). These basins are mostly located in southern Spain and the northern part of Scandinavia, where the natural regime of river flows are affected by human activities like irrigation diversions, hydroelectric power generation, and flood control[26–28]. The current mHM version does not model such heavily human-influenced processes; and the flow from mHM is mostly

representative of the naturalized flow conditions.

The modeled evapotranspiration also shows a good correspondence with the gridded FLUXNET derived ET datasets (Supplementary Figure 15c,d). The median correlation is around 0.97; and 90% of the grid cells exhibit a correlation value of more than 0.76. The relative bias estimated between observed and simulated mean ET is also within a reasonable limit with 80 % of the grid cells having bias between $\pm$ 20 %. While a large number of grid cells covering the study domain show a very good skill in capturing the dynamics of observation based ET, we also found a consistently lower model skill for grid cells located in the drier Mediterranean climate. Notably in these areas the model also have consistently lower skill for the discharge simulations (as indicated above). The poor model performance could be related to some missing processes in mHM and/or errors and uncertainty in the observation based gridded FLUXNET datasets. The gridded FLUXNET dataset used as a reference here is the product of a machine learning algorithm ans was created based on the limited number of underlying eddy flux stations[11].

The agreement between observation based GRACE derived terrestrial water storage (TWS) anomaly and modeled TWS anomaly fields is reasonably good (Supplementary Figure 15e). The median of correlation estimated over all grid cells is around 0.80; and 90% of the grid cells exhibited correlation of more than 0.62. A relatively higher disagreement can be observed in grid cells lying along the Alps and coastal regions. Besides that the model does not account for the glacial and other coastal processes, we note that

the GRACE data have inherently lower fidelity due to larger measurement and leakage errors in accurately quantifying the changes in TWS over these regions[29]. Our analysis results also show that the TWS anomaly have overall the least agreement among the three evaluated variables (Supplementary Figure 15f). These results are consistent and in-accordance with the previous studies for mHM[14,21]; and for other models[30–32].

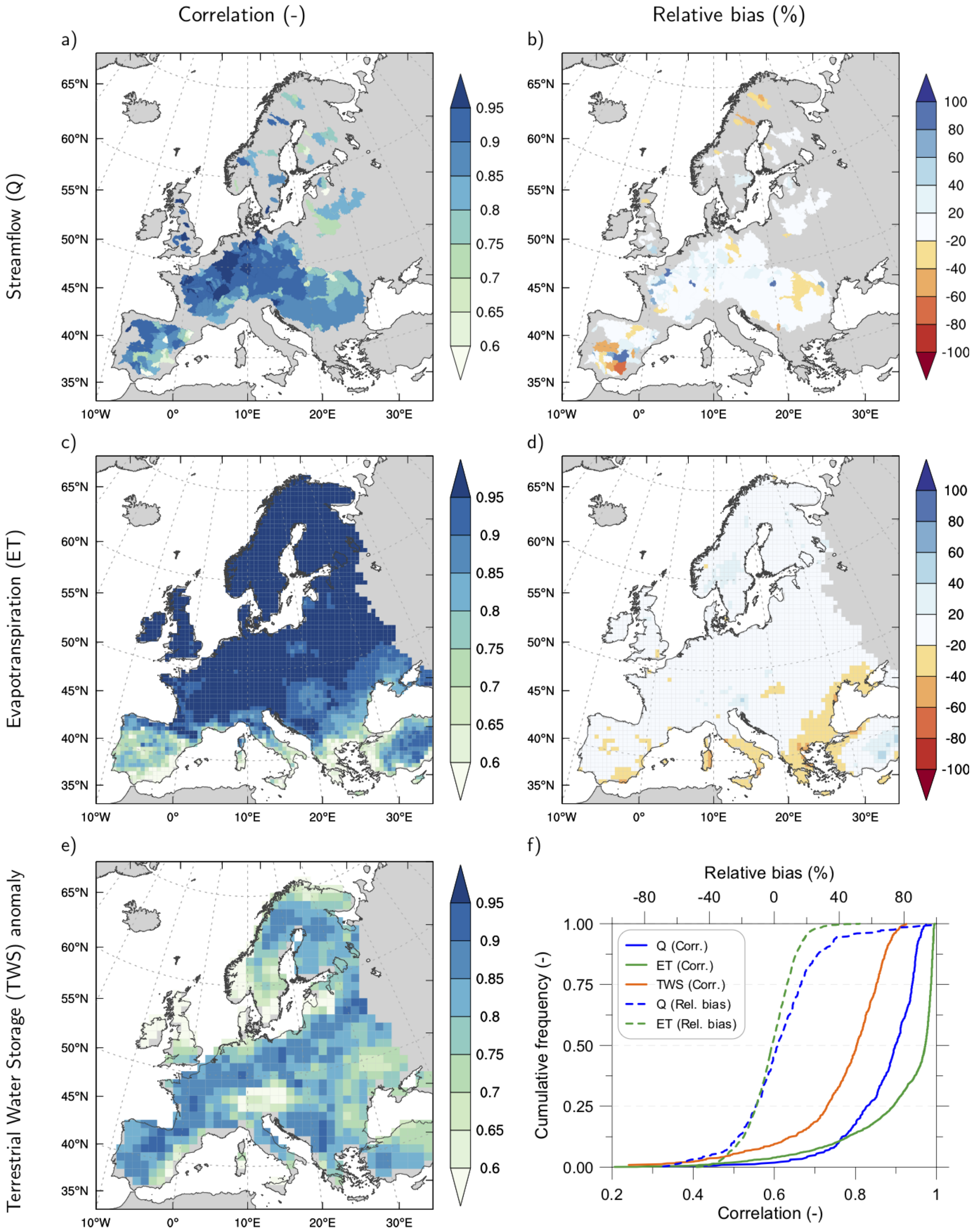

Supplementary Figure 15: Skill of mHM for simulations of monthly streamflow, evapotranspiration and terrestrial water storage (TWS) anomaly across EU in terms of correlation coefficient and relative bias.

1. Hargreaves, G. & Samani, Z. Estimating potential evapotranspiration. *Journal of the Irrigation & Drainage Division - ASCE* **108**, 225–230 (1982).

2. Samaniego, L., Kumar, R. & Attinger, S. Multiscale parameter regionalization of a grid-based hydrologic model at the mesoscale. *Water Resour. Res.* **46** (2010).

3. Kumar, R., Samaniego, L. & Attinger, S. Implications of distributed hydrologic model parameterization on water fluxes at multiple scales and locations. *Water Resour. Res.* **49**, 360–379 (2013).

4. Livneh, B., Kumar, R. & Samaniego, L. Influence of Soil Textural Properties on Hydrologic Fluxes in the Mississippi River Basin. *Hydrol. Processes, Accepted* (2015).

5. EC. Directorate-General for Environment (European Commission). Recommendations for establishing Action Programmes under Directive 91/676/EEC concerning the protection of waters against pollution caused by nitrates from agricultural sources. Part A. Appendix 2, Maps of pedo-climatic zones in Europe. (2011). URL `https://publications.europa.eu/s/m14g`.

6. FAO/IIASA/ISRIC/ISSCAS/JRC. Harmonized World Soil Database (version 1.2), FAO, Rome, Italy and IIASA, Laxenburg, Austria. Tech. Rep., FAO/IIASA/ISRIC/ISSCAS/JRC (2012).

7. West, P. C. *et al.* Leverage points for improving global food security and the environment. *Science* **345**, 325–328 (2014).

8. Samaniego, L. *et al.* Hydrological Forecasts and Projections for Improved Decision-Making in the Water Sector in Europe. *Bulletin of the American Meteorological Society* **100**, 2451–2472 (2019).

9. Samaniego, L. *et al.* Toward seamless hydrologic predictions across spatial scales. *Hydrology and Earth System Sciences* **21**, 4323–4346 (2017).

10. Haylock, M. R. *et al.* A european daily high-resolution gridded data set of surface temperature and precipitation for 1950 – 2006. *J. Geophys. Res* **113** (2008).

11. Jung, M. *et al.* Global patterns of land-atmosphere fluxes of carbon dioxide, latent heat, and sensible heat derived from eddy covariance, satellite, and meteorological observations. *J. Geophys. Res.: Biogeosci.* **116** (2011). G00J07.

12. Landerer, F. W. & Swenson, S. C. Accuracy of scaled grace terrestrial water storage estimates. *Water Resour. Res.* **48** (2012).

13. Sakumura, C., Bettadpur, S. & Bruinsma, S. Ensemble prediction and intercomparison analysis of GRACE time-variable gravity field models. *Geophys. Res. Lett.* **41**, 1389–1397 (2014).

14. Rakovec, O. *et al.* Multiscale and multivariate evaluation of water fluxes and states over European river basins. *J. Hydrometeorol.* **17**, 287–307 (2016).

15. Samaniego, L., Kumar, R. & Zink, M. Implications of parameter uncertainty on soil moisture drought analysis in Germany. *J. Hydrometeorol.* **14**, 47–68 (2013).

16. Molen, W. H. v. d., Martínez Beltrán, J., Ochs, W. J. & Food and Agriculture Organization of the United Nations. *Guidelines and computer programs for the planning and design of land drainage systems* (Food and Agriculture Organization of the United Nations, Rome, 2007).

17. EUROSTAT. European statistics (2016: last accessed Dec, 2019). `https://ec.europa.eu/eurostat/statistics-explained/index.php/Agri-environmental_indicator_-_irrigation`.

18. Wada, Y., Wisser, D. & Bierkens, M. F. P. Global modeling of withdrawal, allocation and consumptive use of surface water and groundwater resources. *Earth System Dynamics* **5**, 15–40 (2014).

19. Sutanudjaja, E. H. *et al.* Pcr-globwb 2: a 5 arcmin global hydrological and water resources model. *Geoscientific Model Development* **11**, 2429–2453 (2018).

20. Swenson, S. C. & Wahr, J. Post-processing removal of correlated errors in GRACE data. *Geophys. Res. Lett.* **33** (2006).

21. Rakovec, O., Kumar, R., Attinger, S. & Samaniego, L. Improving the realism of hydrologic model functioning through multivariate parameter estimation. *Water Resour. Res.* (2016).

22. Zink, M., Kumar, R., Cuntz, M. & Samaniego, L. A high-resolution dataset of water fluxes and states for germany accounting for parametric uncertainty. *Hydrology and Earth System Sciences* **21**, 1769–1790 (2017).

23. Heße, F., Zink, M., Kumar, R., Samaniego, L. & Attinger, S. Spatially distributed characterization of soil-moisture dynamics using travel-time distributions. *Hydrology and Earth System Sciences* **21**, 549–570 (2017).

24. Hanel, M. *et al.* Revisiting the recent european droughts from a long-term perspective. *Scientific Reports* **8**, 9499 (2018).

25. Samaniego, L. *et al.* Anthropogenic warming exacerbates european soil moisture droughts. *Nature Climate Change* **8**, 421 (2018).

26. Batalla, R. J., Gómez, C. M. & Kondolf, G. Reservoir-induced hydrological changes in the Ebro River basin (NE Spain). *J. Hydrol.* **290**, 117–136 (2004).

27. Lorenzo-Lacruz, J., Vicente-Serrano, S., López-Moreno, J., Morán-Tejeda, E. & Zabalza, J. Recent trends in Iberian streamflows (1945–2005). *J. Hydrol.* **414–415**, 463–475 (2012).

28. Arheimer, B., Donnelly, C. & Lindström, G. Regulation of snow-fed rivers affects flow regimes more than climate change. *Nature Communications* **8**, 62 (2017).

29. Jacob, T., Wahr, J., Pfeffer, W. T. & Swenson, S. Recent contributions of glaciers and ice caps to sea level rise. *Nature* **482**, 514–518 (2012).

30. Livneh, B. & Lettenmaier, D. P. Multi-criteria parameter estimation for the unified land model. *Hydrology and Earth System Sciences* **16**, 3029–3048 (2012).

31. Orth, R. & Seneviratne, S. I. Introduction of a simple-model-based land surface dataset for Europe. *Environ. Res. Lett.* **10** (2015).

32. Bai, P., Liu, X. & Liu, C. Improving hydrological simulations by incorporating grace data for model calibration. *Journal of Hydrology* **557**, 291–304 (2018).

Acknowledgements We would like to thank people from various organizations and projects for kindly providing us with the data that were used in this study, which includes USGS, ESA, JRC, NASA, GRDC, BGR and IIASA. We acknowledge the E-OBS dataset from the EU-FP6 project ENSEMBLES (http://ensembles-eu.metoffice.com) and the data providers in the ECA&D project (http://www.ecad.eu). The authors would also like to thank Martin Schrön for his creative art with the mHM conceptualization figure. We would like to thank Uwe Franko for providing the guidance on deriving the organic carbon related denitrification rates and Edwin Sutanudjaja for the help with PCR-GLOBWB model runs. Data analysis was conducted at the High-Performance Computing (HPC) Cluster EVE, a joint effort of both the Helmholtz Centre for Environmental Research-UFZ and the German Centre for Integrative Biodiversity Research (iDiv) Halle-Jena-Leipzig.
